# Recurrent laryngeal inflammatory myofibroblastic tumor with positive anaplastic lymphoma kinase mimicking recurrent respiratory papillomatosis: a case report

**DOI:** 10.1186/1477-7819-12-54

**Published:** 2014-03-06

**Authors:** Chun-yan He, Ge-hong Dong, Hong-gang Liu

**Affiliations:** 1 Department of Pathology, Beijing Tongren Hospital, Capital Medical University, No.1 Dongjiaominxiang Street, Dongcheng District, Beijing 100730, China

**Keywords:** Inflammatory myofibroblastic tumor, Larynx, Pediatric, ALK

## Abstract

Inflammatory myofibroblastic tumor (IMT) of the larynx is an unusual lesion, particularly in the pediatric age group. Laryngeal IMTs in children follow a benign clinical course with reports of only rare recurrences and no metastases. Although anaplastic lymphoma kinase (ALK) has been associated with IMTs, there is only one pediatric laryngeal IMT reported to be ALK-positive with immunohistochemical staining. Here, we present a case of a 10-year-old boy with a laryngeal IMT that recurred four times and was misdiagnosed as recurrent respiratory papillomatosis after the initial three operations. ALK positivity was demonstrated by both immunohistochemical staining and fluorescence *in situ* hybridization. To the best of our knowledge, this case report is the first to describe a laryngeal IMT that recurred multiple times and was confirmed to be ALK-positive at the molecular level.

## Background

Inflammatory myofibroblastic tumor (IMT) is an uncommon lesion that usually involves the lungs and mostly affects young adults [1,2]. In the head and neck region, the paranasal sinuses and orbit are the most commonly affected areas [3]. The laryngeal presentation is extremely rare; about 30 cases have been described in the literature in English and only eight of these involved children [4-11]. Among these eight IMTs, there is only one case of recurrence after resection [7], demonstrating the benign clinical behavior of this tumor. In addition, although anaplastic lymphoma kinase (*ALK*) gene translocation can be seen in about 50% of IMTs in young patients [2,12], there is only one case of childhood laryngeal IMT that reported ALK-1 positivity by immunohistochemical staining [4]. So far, there are no reports of *ALK* gene translocation detected at the molecular level in laryngeal IMTs.

Here, we present a case of a laryngeal IMT in a 10-year-old boy. It recurred four times and was misdiagnosed as recurrent respiratory papillomatosis (RRP) after the initial three operations. ALK positivity was demonstrated by both immunohistochemical staining and fluorescence *in situ* hybridization (FISH). Despite the excellent prognosis of laryngeal IMTs, this case presented a clinical and surgical challenge in the form of multiple recurrences, due mainly to incomplete tumor resection.

### Case presentation

**Figure 1:**
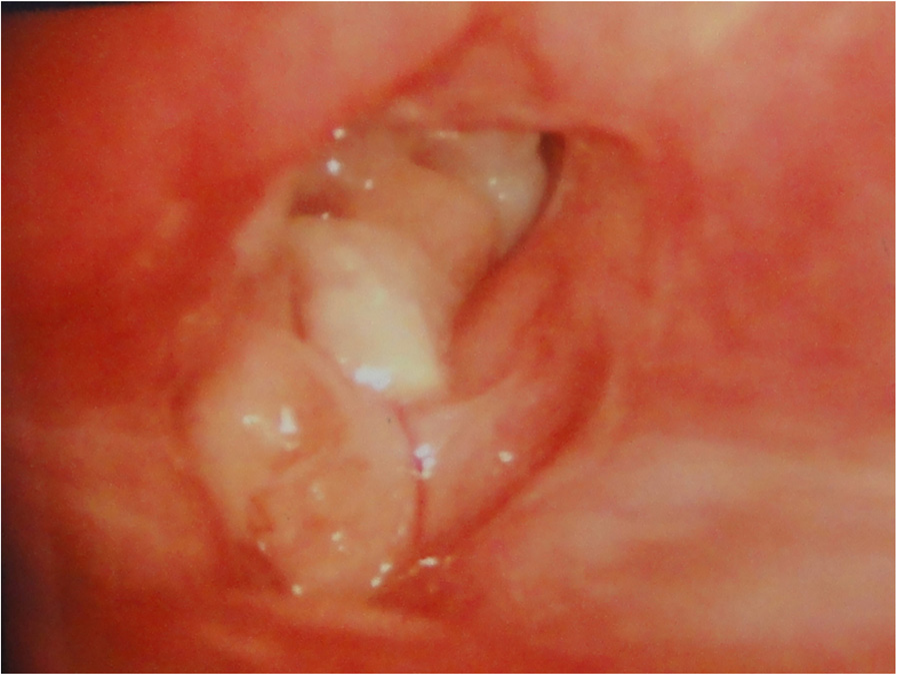
**Laryngoscopic view.** Exophytic masses involved the whole length of the right vocal cord and reached the anterior commissure and front of the left vocal cord.

A 10-year-old boy was referred to a local hospital with hoarseness for 1 year in July 2009. Clinicians saw a tumor of the vocal cord, which subsequently was resected by using direct microlaryngoscopy with a carbon dioxide (CO_2_) laser. Respiratory papilloma was diagnosed after pathologic analysis. After the procedure, the symptom of hoarseness actually worsened and the patient also gradually developed a foreign body sensation. He then presented to our hospital in May 2010. Fibrolaryngoscopy demonstrated exophytic masses involving the whole length of the right vocal cord and extending to the anterior commissure and anterior third of the left vocal cord (Figure 1). The cords were mobile, and no cervical lymphadenopathy was appreciated on palpation. In addition, there was no recent history of fever or weight loss, and laboratory findings were normal. The tumor was resected by using direct microlaryngoscopy with a CO_2_ laser, and histological examination was performed (see below).

Six months after the second operation, the patient was referred again for hoarseness in addition to shortness of breath. Via laryngoscopy, similar exophytic masses were observed at the same location, and these masses were then resected. However, the lesion returned 5 months later and was removed again, only to reappear a fourth time after 2 months. The tumor from the fourth operation showed a proliferation of spindle cells in the subepithelial stroma. A diagnosis of IMT was made after reviewing the histopathology, combined with the results of immunohistochemical staining and testing for human papillomavirus (HPV) and ALK. During a clinical and pathological discussion, it was proposed that incomplete resection was the cause of tumor relapse. A fifth operation was performed, and frozen sections of the surgical margins sent at the time of the procedure were all negative for IMT. The child’s voice improved after 3 months, and the tumor failed to recur during 2 years of regular follow-up. This report was approved by the hospital ethics committee, and written informed consent for publication of the clinical details and clinical image was obtained from the patient’s guardian.

**Figure 2:**
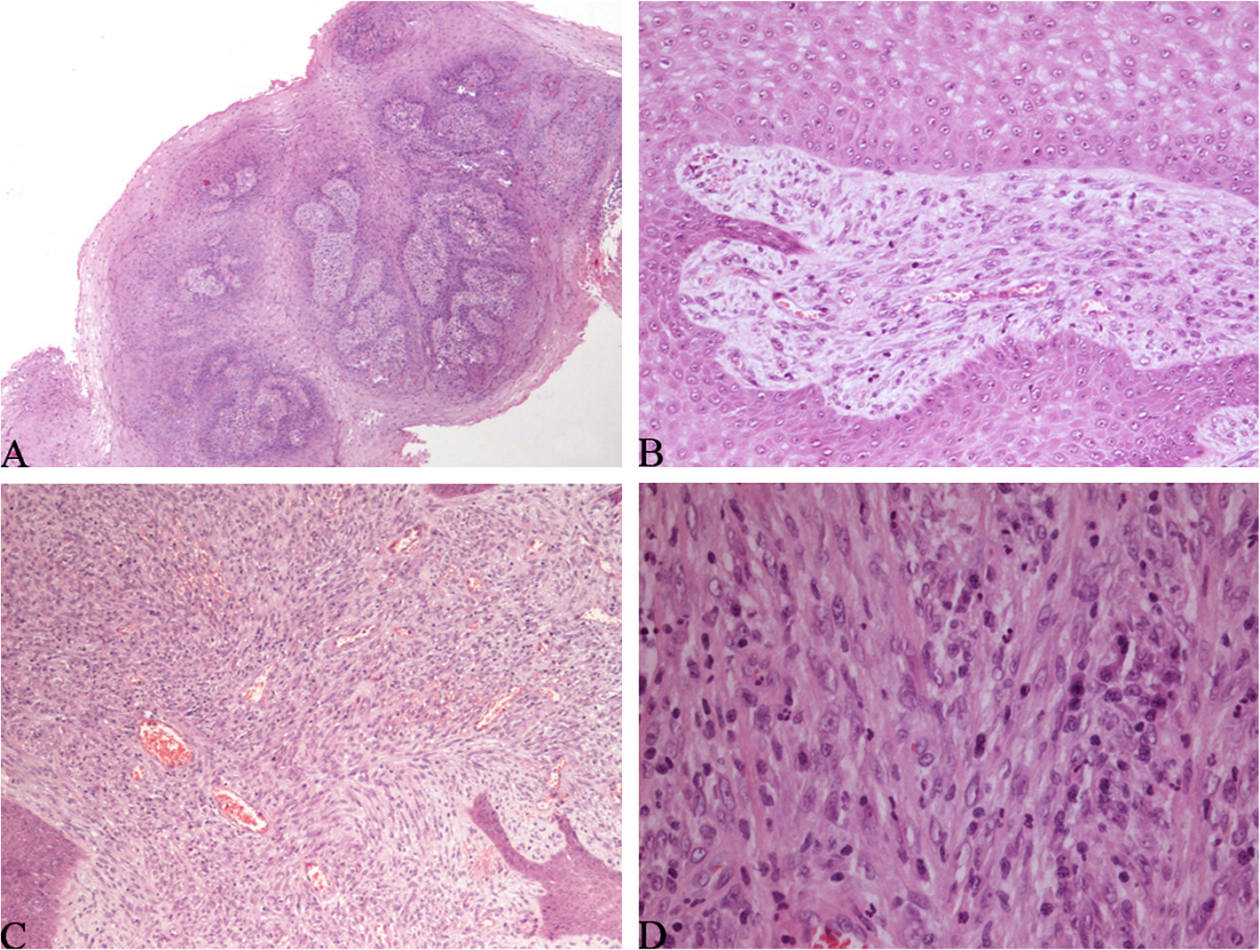
**Histological findings. (A)** The lesions displayed a papillary and polypoid appearance (hematoxylin-eosin [HE] × 40). **(B)** There was obvious spindle cell hyperplasia in the fibrovascular cores (HE × 200). **(C)** The spindle cells were arranged in both a fascicular and storiform pattern with lymphocytes and plasma cells in the background (HE × 100). **(D)** The spindle cells contained pale, eosinophilic cytoplasm and elongated nuclei with one or more small nucleoli (HE × 400).

**Figure 3:**
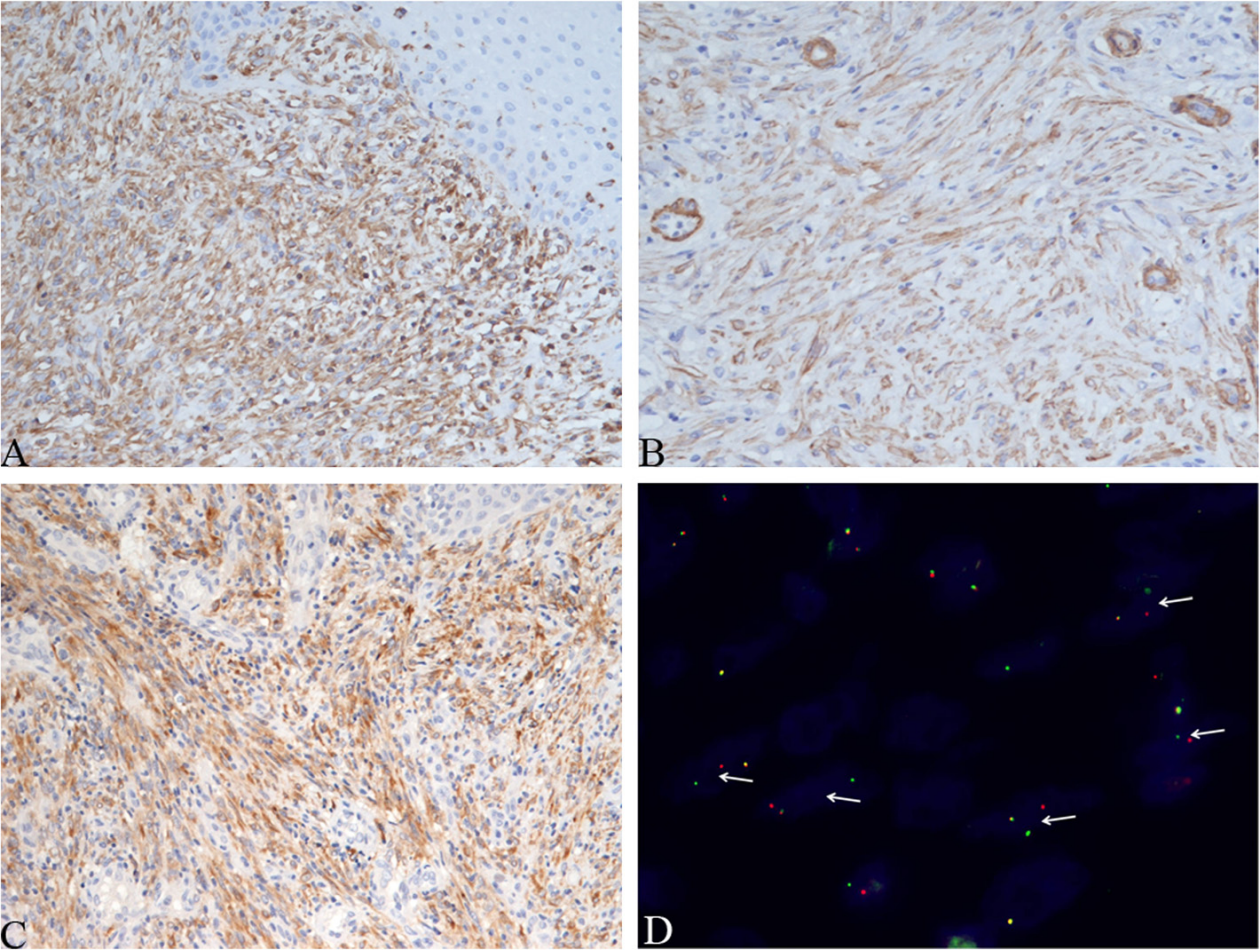
**Immunohistochemical findings and fluorescence *in situ *hybridization (FISH) studies.** The spindle cells were positive for vimentin **(A)**, α-smooth muscle actin **(B)**, and anaplastic lymphoma kinase-1 (ALK-1) **(C)** (immunohistochemistry staining × 200). **(D)** FISH studies demonstrated rearrangements of the *ALK* gene with split of the red and green signals (arrows) in the spindle cells.

All histologic sections from the five operations were reviewed. Because the primary lesion and the recurrences had similar histological and immunohistochemical features, they are described together. Microscopically, the lesions displayed an exophytic, polypoid, and papillary appearance under low power (Figure 2A) with a hyperplastic squamous epithelial lining. In this squamous lining, there appeared to be koilocyte-like cells. The subepithelium consisted of fibrovascular cores. Most of the cores were large and compact with obvious hyperplasia of spindle cells (Figure 2B), unlike the fibrovascular cores of squamous papillomas, which are thin and loose. The hyperplasia of stromal cells was more obvious in the last two specimens because the sections from the first three operations were relatively superficial. The spindle cells were arranged in both a fascicular and storiform pattern with lymphocytes and plasma cells in the background (Figure 2C). The spindle cells contained pale, eosinophilic cytoplasm and nuclei that were elongated and slightly pleomorphic with one or more small nucleoli (Figure 2D). Occasional regular mitoses (about 0.8 per 10 high-powered fields) and some ganglion-like cells could be seen, and necrosis was absent. Immunohistochemically, the squamous epithelium was negative for P16. The spindle cells were positive for vimentin, smooth muscle actin, and ALK-1 (Figures 3A-C) but negative for desmin, muscle-specific action, S-100, Bcl-2, CD34, cytokeratin, and p53. The Ki-67 proliferation index was about 3%.

We applied *in situ* hybridization as well as polymerase chain reaction analysis to test for the HPV genotype. The two analyses included HPV types 6, 11, 16, 18, 31, 33, 35, 39, 42, 43, 44, 45, 51, 52, 53, 58, 59, 66, 68, and CP8304. The viral tests did not demonstrate any of the above-mentioned HPV types. We performed FISH with Vysis LSI ALK Dual-Color, Break-Apart Rearrangement Probe (Abbott Molecular, Abbott Park, IL, USA) on the spindle cells and found translocations involving the *ALK* locus (Figure 3D). On the basis of the histopathological and immunohistochemical features and the result of the FISH test, a diagnosis of IMT was rendered.

## Discussion

IMT involving the larynx is rare, especially in the pediatric age group. To date, only eight cases of childhood laryngeal myofibroblastic lesions have been reported in the literature in English: four cases diagnosed as IMT and four as inflammatory pseudotumor (IPT) [4-11]. The mean age of these patients is 5.7 years (range of 2.5 to 10 years), and the male-to-female ratio is 7:1. Dyspnea and stridor are the most frequently reported symptoms, whereas hoarseness (which is more common in adult laryngeal IMTs) is seen in only two cases, who are relatively older. The mean symptom duration is 3.9 months (range of 0.5 to 6 months) [4-11]. Most cases generally involve the subglottic region, and only one involved the vocal cord. The majority of the lesions are nodular or polypoid, and one case mimicked papillomatosis [5]. The lesions rarely recur after tumor resection, showing benign behavior, except for one report of a single recurrence after an incomplete resection [7]. Our patient was a 10-year-old boy with hoarseness for 1 year. The lesion was located in the vocal cord, had a polypoid and papillary appearance, and recurred four times. We believe that the relatively superficial resections of the initial surgeries and the wrong pathological diagnosis were the reasons behind the multiple recurrences. The histological features that led to the incorrect diagnosis of papilloma included papillary growth, squamous epithelial hyperplasia with mild atypia, and koilocyte-like cells within the epithelium (although results of HPV-related tests were negative). The clinical diagnosis of RRP led the otolaryngologists to neglect the surgical margins.

The etiology of IMT is still controversial. Viruses that are suspected to be involved in the development of IMT include human herpesvirus-8 (HHV-8) and Epstein-Barr virus (EBV), as reported in cases of adult pulmonary, splenic, and hepatic IMTs [13,14]. There is no report of HHV-8- or EBV-positive laryngeal IMTs in adults or children. IgG4-related disease is a recently described multisystem disorder [15] that may also be associated with IMT. The disease is characterized histologically by an infiltrate of plasma cells, which is similar to inflammatory myofibroblastic tumors [16]. However, only a single IgG4-positive laryngeal IMT has been reported (in a 56-year-old man) [17] and none in children. A physical cause of laryngeal IMT has been proposed by multiple reports. Trauma or potential subclinical traumatic stimuli such as voice abuse, excessive coughing, and acid-reflux are often associated with wound healing, which includes the presence of myofibroblasts. Several laryngeal IMT reports suggest that trauma or subclinical trauma may be one etiology of IMT [17-19]. However, there are scholars who argue that trauma-induced myofibroblastic proliferative lesions should be diagnosed as IPT, which is a reactive hyperplasia, rather than IMT—a true neoplasm [20]. The terms IPT and IMT of the larynx are still used interchangeably in the literature to describe a fibroblastic/myofibroblastic spindle cell proliferation with admixed inflammatory cells [2,3,14,18]. According to the World Health Organization’s classification of tumors, the definition of IMT is a distinct borderline lesion composed of myofibroblastic cells with a variable admixture of inflammatory cells, and the terms IPT and IMT are considered synonymous [21]. It is difficult to diagnose an IPT or IMT because the lesions with the above-mentioned histology exhibit highly variable and unpredictable clinical behavior [12].

Identification of chromosomal translocations of the *ALK* gene located on chromosome 2p23 supports the hypothesis that IMTs are neoplastic in origin. Approximately 50% of IMTs show ALK positivity by FISH or immunohistochemistry or both [12]. The ALK-positive rate is reported slightly more frequently by immunohistochemical staining than FISH [22]. In addition, ALK expression in IMT is more common in younger patients [12]. However, among the reported pediatric laryngeal IMTs, only one showed immunoreactivity for ALK-1 protein [4]. In our case, both immunohistochemical and FISH analyses were positive for ALK protein expression and translocations involving the *ALK* locus, respectively. So far, our case is the only laryngeal IMT that was confirmed to have the *ALK* gene translocation at the molecular level.

ALK positivity confirmed that the IMT in our case was neoplastic. Could this have been the cause of the repeated tumor recurrence in our patient? We are not certain. The laryngeal IMT case reports followed a benign clinical course with rare local recurrences and were associated with a low rate of ALK positivity. Those IMTs that are negative for ALK may not be true neoplasms and thus have a better prognosis. However, among the children’s laryngeal IMT reports, the only ALK-positive case had no recurrence, and the one recurrent case was ALK-negative [4,7]. In other anatomic locations, there are many reports of ALK-negative IMTs showing invasive biological behavior [23-25]. Accordingly, we think that complete resection of the tumor with negative surgical margins is the key to preventing recurrence. In the last operation for our case, frozen sections were performed to ensure negative surgical margins, and there has been no recurrence for 2 years.

## Conclusions

Pediatric laryngeal IMT is extremely rare. We present the first case of a pediatric laryngeal IMT with multiple recurrences and ALK positivity confirmed by FISH. Although most laryngeal IMTs have benign clinical behavior, complete surgical resection with negative tissue margins and frequent, regular follow-ups are necessary because the lesion has unclear pathogenesis and the capacity for recidivism.

## Abbreviations

ALK: Anaplastic lymphoma kinase; EBV: Epstein-Barr virus; FISH: Fluorescence in situ hybridization; HE: Hematoxylin-eosin; HHV-8: Human herpes virus-8; IHC: Immunohistochemistry; IMT: Inflammatory myofibroblastic tumor.

## Competing interests

The authors declare that they have no competing interests.

## Authors’ contributions

All authors participated in the conception and design of the study. CY He performed the clinical analysis and drafted the manuscript. GH Dong performed the experiments and prepared the figures. HG Liu supervised the study. All authors read and approved the final manuscript.
